# Silica exposure increases the risk of stroke but not myocardial infarction—A retrospective cohort study

**DOI:** 10.1371/journal.pone.0192840

**Published:** 2018-02-26

**Authors:** Chenjing Fan, Pål Graff, Per Vihlborg, Ing-Liss Bryngelsson, Lena Andersson

**Affiliations:** 1 Department of Occupational and Environmental Medicine, Faculty of Medicine and Health, Örebro University, Örebro, Sweden; 2 National Institute of Occupational Health (STAMI), Oslo, Norway; Medical University Innsbruck, AUSTRIA

## Abstract

**Introduction:**

Work-related exposure to silica is a global health hazard that causes diseases such as silicosis. Some studies have also reported that silica exposure is linked to elevated cardiovascular disease mortality. However, these diagnoses have not been investigated in detail and there have been few studies on morbidity. The aim of this study is to examine morbidity and mortality from different cardiovascular diseases among silica-exposed Swedish foundry workers.

**Methods:**

Historical and contemporary measurements (1968–2006) of respiratory silica exposure were matched to job categories, individual foundries, and 4 time periods (1968–1979, 1980–1989, 1990–1999, 2000–2006) using a mixed model. Morbidity and mortality data for the studied cohorts were matched against the General Population Registry. Statistical analyses were performed with SPSS and STATA, and the data were stratified by age, gender, and year.

**Results:**

Mortality from cardiovascular disease (SMR 1.3; 95% CI 1.2–1.4) and stroke (SMR 1.6, 95% CI 1.2–2.1) was significantly elevated among the studied population. The cohort also exhibited significantly elevated morbidity from stroke (SIR 1.34; 95% CI 1.2–1.5) but not myocardial infarction. The mean age at the time of first morbidity from stroke was 64 years, with 36% of the cases occurring before the age of 60.

**Conclusions:**

Swedish foundry workers exposed to respirable silica exhibit elevated morbidity and mortality from stroke, but not from myocardial infarction. Our results also suggest a relationship between silica exposure and morbidity from stroke at a younger age than the general population.

## Introduction

Occupational silica exposure is a global health issue. It is well established that work-related silica exposure is associated with elevated mortality because of respiratory diseases such as silicosis [1–3]. Measures to reduce silica inhalation based on structural, environmental and technical improvements in workplaces have greatly reduced the incidence of silicosis[4]. However, mortality and morbidity from silicosis among workers at risk of occupational silica exposure in Sweden remain significantly higher than among the general population. Furthermore, several epidemiological studies have linked silica exposure to elevated mortality from cardiovascular disease (CVD) as well as cancers and renal dysfunction[5–9]. However, statistical analyses focusing on specific CVD types have yielded inconsistent results.

Silica is often present at industrial sites in one or more of its crystalline forms—quartz, cristobalite and tridymite. The latter two forms are created during high-temperature processes. Crystalline silica typically exists as respirable particles, and silica nanoparticles (i.e. particles smaller than 100nm) can enter the bloodstream [10–12]. Particles that can enter the respiratory system during breathing are divided into inhalable, thoracic and respirable particles, which are potentially harmful to health if deposited in the lungs or airways. Their precise toxicity depends on their physicochemical properties [13].

Occupation related silica-exposure has been related to increased risks of (and mortality from) diseases including silicosis, lung cancer, renal disease, and CVD for particulate silica exposure[5–9, 14]. However, the available data for specific categories of CVD are inconsistent. Cohort studies have suggested that an apparent silica exposure-related decrease in the risk of ischemic heart disease (IHD) might be due to the healthy worker effect [15].

Respirable silica is encountered in many kinds of industrial sites, including foundries. Three kinds of foundry exist in Sweden: iron, steel and metal foundries. The general production processes in foundries are sand mixing, core making, moulding, melting, casting, shake out, and fettling. The Swedish foundry workers with the highest overall silica exposure levels are sand mixers, fettlers, and furnace and ladle repair workers; those with the lowest exposures are casters and core makers [4, 16]. The Occupational Exposure Limit for silica in Sweden has been 0.1mg/m^3^ since 1979, but 8% of silica-exposed workers exceed this limit [17].

As mentioned above, the negative health effects of work-related silica-exposure are well-established despite the healthy worker effect [15]. However, to our knowledge, few studies on the adverse health effects of silica have primarily focused on CVD, even though many have included various CVD diagnoses in their analyses and found surprising elevations. Most studies also examine mortality alone; few look at it in conjunction with morbidity. CVD is a major cause of death worldwide, and there is a need for more information about its relationship to work-related silica-exposure. Therefore, this study analyses the relationship between silica exposure in Swedish foundries and stroke and myocardial infarction with respect to mortality and morbidity.

## Material and methods

### Measurement database

Silica measurements used in this study include both recently obtained data and historical measurements from the examined foundries. The recently obtained data consist of 340 silica measurements performed at the studied foundries by the Department of Occupational and Environmental Medicine at Örebro University Hospital between April 2005 and May 2006. Historical measurement data acquired between 1968 and 2004 were obtained from compulsory measurements performed by the foundries themselves and the National Exposure survey 1968–1974, which was performed by the Swedish Work Environment Authority. The sampling method used in the recent measurements was chosen to ensure comparability with the historical data, which were corrected to facilitate this. Measurement times range from 240–600 minutes for recent data and were corrected to 8 hour time-weighted average concentrations (8-hour TWA), representing a full workday.

A total of 1,667 measurements (1,327 historical and 340 recent) were obtained from the 10 foundries included in this study. Because we have no measurements acquired before 1968, exposure times before this year were estimated to be identical to the corresponding average values for the period 1968–1979. The job categories used in the measurement datasets were caster, core maker, fettler, furnace and ladle repair, maintenance, melter, moulder, sand mixer, shake out, transportation, other specified, many jobs, foundry worker, and other unspecified. ‘Other specified’ includes cleaners, painters and model carpenters. ‘Many jobs’ includes subjects who performed multiple specified foundry jobs. ‘Foundry workers’, were employees with no specified working assignments, and ‘other unspecified’ employees were those without job titles. The exposure values for the ‘other specified’ and ‘foundry worker’ groups were assumed to be equal to the mean exposure for all the other groups. The mean exposure for the ‘other unspecified’ and ‘many jobs’ groups was estimated as the mean exposure for all other groups with specified job titles.

### Cohort

**Fig 1:**
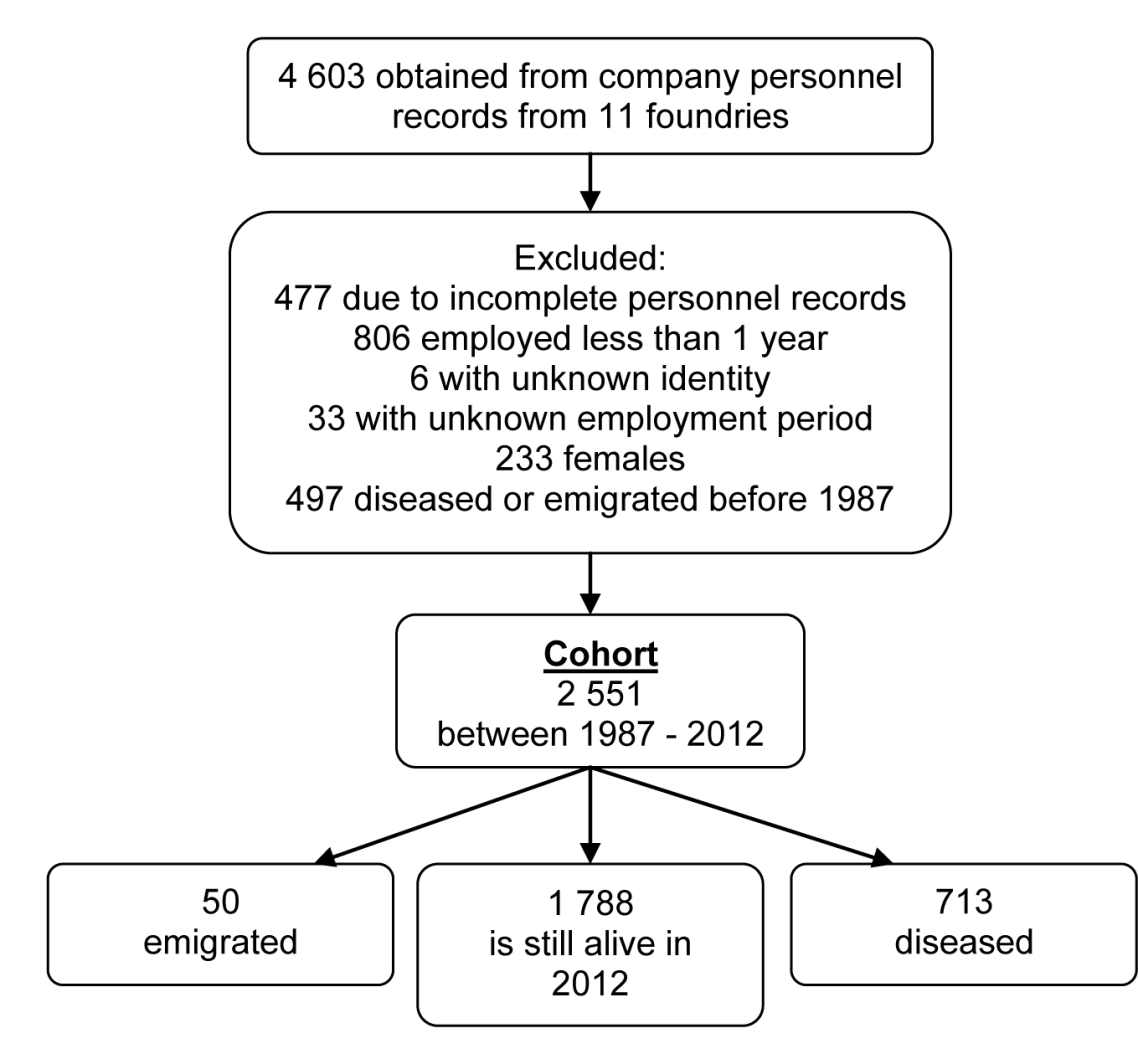
Cohort selection process showing the exclusion criteria and the number of subjects considered for inclusion when examining mortality (n = 3048) and morbidity (n = 2551). Subjects were followed until the year 2012 or the year they became diseased or emigrated, whichever came first.

Company personnel records of workers employed between 1913–2005 were obtained from 11 Swedish foundries that differ in size, sand usage, and the moulds, cores, and techniques that they employ. A total of 1555 workers were excluded (Fig 1). Exclusion criteria were: having been employed at the foundry for less than 1 year (n = 806), unknown identity (n = 6), unclear employment duration (n = 33), an incomplete personnel record (n = 477), and female gender (n = 233). Individuals who diseased or emigrated before 1987 (n = 497) were also excluded, leaving 2551 in the cohort (Fig 1). Subjects were followed until the year 2012 or the point at which they became diseased or emigrated, whichever came first.

The number of morbidity cases was also analyzed per cumulative silica exposure quartile (<0.11, 011–0.25, 0.25–0.65, >0.65) and as a function of employment length. In these analyses, cases of CVD were classified as either MI (ICD10: I21-I22) or stroke (ICD10: I61, I63, I64).

### Statistical analysis

Using a previously reported approach[9, 16], a mixed model was constructed to estimate the silica exposure of foundry employees over four time periods (1968–1979, 1980–1989, 1990–1999, 2000–2006), 10 foundries, and 11 job categories (as well as three groups of workers with no formal job title). To compensate for the skew of the measured silica levels, the measurement data were log-transformed before further analysis.

The model made it possible to identify factors affecting silica exposure and to estimate representative cumulative silica exposure values (in mg/m^3^ * years) for individual workers based on their employment duration, job title, the foundry they worked at and the period(s) of time during which they worked there, and the duration of their exposure. The initial estimates produced by the model were subjected to antilogarithmic transformation to yield estimated silica exposures for different time periods, jobs, and foundries. Cumulative exposures were calculated for each worker included in the study; exposures before 1968 were estimated to be equal to the corresponding average exposure for 1968–1979.

### SMR/SIR

Mortality and morbidity were assessed using the standardized mortality ratio (SMR) and standardized incidence ratio (SIR), respectively. Data on the incidence of CVD were retrieved from the Swedish National Patient Register of the National Board of Health and Welfare, which was established in 1987. Therefore, information could only be retrieved for employees who were alive in 1987. Mortality data were retrieved from the Swedish Social Service mortality registers, which were also established in 1987.

Everyone resident in Sweden is assigned a unique personal identification number, and Sweden offers its residents universal access to healthcare and hospital services. Hospitals are required to record and register diagnoses made after appointments or upon discharge. The Swedish National Patient Register has a >99% coverage of hospital diagnoses from 1987 onward, and a positive predictive value of 85–95%, making it an excellent tool for epidemiological studies because it allows researchers to retrieve morbidity and mortality data for the entire patient population [18]. Morbidity and mortality data for the studied cohorts were matched against the General Population Registry. Statistical analyses were performed with SPSS version 22 and STATA version 14.0.

Stratifications by age, gender and year were performed with STATA. It has not been possible to obtain subject information about other known and possible effectors of CVD morbidity among foundry workers such as smoking, shift work, noise, and carbon monoxide (CO) exposure [19–21]. The potential confounding effects of these factors are discussed in the ‘Discussion and conclusion’ section.

## Results

The cohort examined in this study consists of 2551 individuals (Fig 1). General information was obtained by using SPSS to map their distribution across job categories, years of employment, and first year of employment, and to obtain information on cohort characteristics such as birth year and cumulative exposure (Table 1). The results presented in Table 1 show that most workers in the cohort began working at the foundries in 1970–1989 and worked there for 1–4 years or 5–9 years. The most common job category was fettler, which (as noted previously) was one of the groups with the highest exposure to respirable silica. The oldest worker in the cohort was born in 1897, but the mean and median years of birth were 1948 and 1950, respectively. Moreover, the calculated standard deviations and ranges of the age at start of employment and years of employment demonstrate that both these variables were widely spread. However, most of the cohort were in their 20s when they began working at the foundries.

**Table 1:** General demographic information on the cohort used in this study.

		Number of subjects		Percent*	
Start of work (year)					
1910–1929		2		0,1	
1930–1949		167		6,5	
1950–1969		586		23,0	
1970–1989		1139		44,6	
1990-		657		25,8	
Years of employment					
1–4		858		33,6	
5–9		541		21,2	
10–14		333		13,1	
15–19		236		9,3	
>20		583		22,9	
Job categories					
Many jobs		325		12,7	
Caster		78		3,1	
Moulder		236		9,3	
Core maker		268		10,5	
Sand mixer		7		,3	
Melter		150		5,9	
Furnace and ladle repair		7		,3	
Shake out		22		,9	
Fettler		486		19,1	
Maintenance		187		7,3	
Transportation		32		1,3	
Other specified		104		4,1	
Foundry workers		263		10,3	
Other unspecified		386		15,1	
Total		2551		100,0	
	Mean	Median	Standard Deviation	Minimum	Maximum
Birth year	1948	1950	18,8	1897	1985
Age at start of employment	28	25	10,3	12	74
Years of employment	11,7	7,3	11,0	1,0	54,6
Exposure for silica mg/m^3^**	0,48	0,26	0,58	0,015	10,3

*percent of total cohort (n = 2551).

** Cumulative respirable silica-exposure

Statistically significant increases in SMR were seen for total mortality (SMR 1.3; 95% CI 1.2–1.4), CVD (SMR 1.4; 95% CI 1.3–1.6) and stroke (SMR 1.6, 95% CI 1.2–2.1) (Table 2). Additionally, the morbidity analysis showed that the cohort exhibited a significantly increased incidence of stroke (SIR 1.34; 95% CI 1.2–1.5) but not myocardial infarction (Table 3). Further stratification by silica exposure quartiles and employment length revealed no dose-response behavior for either MI or stroke, but the number of observed CVD cases was significantly greater than the number of expected cases among some foundry worker classes. The mean age at the point of first morbidity from stroke was 64, but 36% of first morbidities occurred below the age of 60.

**Table 2:** Analysis of mortality (SMR) in the cohort. SMR values are shown for total mortality, cardiovascular disease, acute myocardial infarction and stroke.

	obs	exp	SMR	95% CI
Total mortality	713	548.8	1.30	1.21–1.40
Cardiovascular disease	338	239.8	1.41	1.26–1.57
Acute myocardial infarction	100	136.2	0.73	0.60–0.89
Stroke	47	29.3	1.61	1.18–2.14

**Table 3:** Analysis of morbidity (SIR) due to either myocardial infarction or stroke in the cohort. Results are shown for the whole cohort and the cohort stratified by cumulative silica-exposure quartiles and employment length.

			Obs	Exp	SIR	95% CI
Myocardial	Totalt		311	309.6	1.00	0.9–1.1
Infarction						
ICD10: I21-I22						
	Quartz quartiles	<0.11	36	36.1	0.99	0.7–1.4
		0.11–0.25	75	57.3	1.31	1.0–1.6
		0.25–0.65	67	80.4	0.83	0.7–1.1
		>0.65	133	135.8	0.98	0.8–1.2
	Employment time(in years)	< = 2	36	36.2	0.99	0.7–1.4
		2.1–10	102	96.3	1.06	0.9–1.3
		10.1–20	73	69.8	1.05	0.8–1.3
		>20	100	107.2	0.93	0.8–1.1
Stroke	Totalt		327	243.5	1.34	1.2–1.5
ICD10: I61, I63, I64						
	Quartz quartiles	<0.11	38	26.8	1.42	1.0–2.0
		0.11–0.25	64	43.3	1.48	1.1–1.9
		0.25–0.65	66	62.4	1.06	0.8–1.4
		>0.65	159	111.0	1.43	1.2–1.7
	Employment time(in years)	< = 2	43	27.4	1.60	1.1–2.1
		2.1–10	93	74.3	1.25	1.01–1.5
		10.1–20	73	55.3	1.32	1.04–1.7
		>20	118	86.6	1.36	1.1–1.6

## Discussion

In this study we analyze the relationship between work-related silica exposure and CVD. Our main conclusion is that Swedish foundry workers have a higher overall mortality from CVD and stroke than the general population (Table 2), but not from MI. This group also has a higher morbidity from stroke than the general population (Table 3). The apparent relationship between CVD and respirable silica exposure may be due to silica-induced inflammation [22].

Two previous studies found a statistically significant reduction in stroke mortality among individuals exposed to respirable silica,[23, 24] while a third found non-significantly elevated mortality among such individuals [7]. Our study is thus, to our knowledge, unique in presenting a statistically significant elevation of stroke mortality and morbidity among silica-exposed workers.

Our results show that the mean age at first morbidity from stroke was 64 years. The corresponding mean age for the general population in Sweden is higher, with only 14% of all cases occurring before the age of 60 (based upon the Swedish National Patient Register, 2008). Conversely, 36% of the cases among our cohort occurred before the age of 60, suggesting that silica-exposed foundry workers tend to suffer strokes at a younger age than the general population.

A limitation of this study is the lack of data on potential confounding factors that could affect the SMR/SIR results, which is a common problem in epidemiological studies like this. Although we stratified by age, gender, and year in our statistical analysis, we could not obtain any information on other confounding factors such as the subjects’ eating habits, physical activity, noise exposure, shift working patterns, smoking habits, or carbon monoxide (CO) exposure.

Smoking is a known risk factor for CVD [21] and is often seen as a confounder in epidemiological studies like this. However, its actual impact on study results is unclear.[21] A study on occupation-related particulate air pollution and the risk of CVD mortality that examined a large cohort of Swedish foundry workers found no significant differences between smokers and the whole cohort [25].

Prolonged exposure to industrial noise (≥85dB) and shift work (i.e. any pattern of working hours other than weekdays between 06:00 and 18:00) have been linked to an increased risk of hypertension,[20] which is an established risk factor for atherosclerosis and CVD. Noise is also associated with MI, stroke and coronary heart disease [20]. However, there are some inconsistencies in the data supporting this relationship, which have been attributed to methodical differences [26].

We observed no dose-response relationship between silica exposure and morbidity. However, a study published in 2014 reported a relationship between ‘low-level silica-exposure’ (0.76–1.84 mg/m^3^) and elevated mortality from total heart disease and IHD [5]. The authors speculated that the reduced mortality from heart disease and IHD among highly exposed individuals may be due to increased rates of mortality due to respiratory and pulmonary diseases; this may also explain the seemingly contradictory results of other studies on the relationship between silica exposure and CVD. The ‘low level silica-exposure’ range used in that work was actually higher than the highest quartile considered in this study (>0.65 mg/m^3^; Table 3). We thus believe that such differences between the silica-exposure levels considered in earlier studies may have contributed to some of the inconsistencies in the literature on the relationship between silica exposure and CVD.

### Conclusion

We have analyzed the relationship between work-related silica-exposure and CVD. Our main conclusion is that compared to the general Swedish population, Swedish foundry workers have a higher overall mortality from CVD and stroke but not from myocardial infarction. These workers also exhibit elevated morbidity from stroke but not from myocardial infarction.

## Ethical considerations

This project was approved by the Uppsala ethical review board; its registration number is 2004:M-374.

## Abbreviations

CHD: coronary heart disease
CO: carbon monoxide
CVD: cardiovascular disease
IHD: ischemic heart disease
MI: Myocardial infarction
